# Traditional Knowledge, Phytochemistry, and Biological Properties of *Vachellia tortilis*

**DOI:** 10.3390/plants11233348

**Published:** 2022-12-02

**Authors:** Douae Taha, Souad El Hajjaji, Yassine Mourabit, Abdelhakim Bouyahya, Learn-Han Lee, Naoual El Menyiy, Aanniz Tarik, Taoufiq Benali, Hamza El Moudden, Monica Gallo, Naima Iba, Ilhame Bourais

**Affiliations:** 1 Laboratory of Spectroscopy, Molecular Modeling Materials, Nanomaterials Water and Environment—CERNE2D, Faculty of Sciences, Mohammed V University in Rabat, Rabat 10100, Morocco; 2 Laboratory of Human Pathologies Biology, Department of Biology, Faculty of Sciences, Genomic Center of Human Pathologies, Mohammed V University in Rabat, Rabat 10106, Morocco; 3 Novel Bacteria and Drug Discovery Research Group (NBDD), Microbiome and Bioresource Research Strength (MBRS), Jeffrey Cheah School of Medicine and Health Sciences, Monash University Malaysia, Bandar Sunway 47500, Malaysia; 4 Laboratory of Pharmacology, National Agency of Medicinal and Aromatic Plants, Taounate 34025, Morocco; 5 Medical Biotechnology Laboratory (MedBiotech), Rabat Medical & Pharmacy School, Mohammed V University in Rabat, Rabat 10100, Morocco; 6 Laboratory of Natural Resources and Environment, Polydisciplinary Faculty of Taza, Sidi Mohamed Ben Abdellah University of Fez, Taza-Gare, Taza 30050, Morocco; 7 Higher School of Technology of El Kelaa Des Sraghna, Cadi Ayyad University, El Kelaa Des Sraghna BP 104, Marrakesh 40001, Morocco; 8 Department of Molecular Medicine and Medical Biotechnology, University of Naples Federico II, Via Pansini 5, 80131 Naples, Italy

**Keywords:** *Vachellia tortilis*, *Fabaceae* family, bioactive compounds, medicinal use, antimicrobial activities, pharmacological effects

## Abstract

*Vachellia tortilis* is a medicinal plant of the Fabaceae family, widely distributed in arid and semi-arid regions of North, East and Southern Africa, the Middle East and the Arabian Peninsula. In traditional medicine. It’s commonly used to treat certain ailments, including diabetes, asthma, hepatitis and burns. Different scientific search databases were used to obtain data on *V. tortilis*, notably Google Scholar, Scopus, Wiley Online, Scifinder, Web of Science, ScienceDirect, SpringerLink, and PubMed. The knowledge of *V. tortilis* was organized based on ethnomedicinal use, phytochemistry, and pharmacological investigations. Phytochemical studies revealed the presence of a variety of phytocompounds, including fatty acids, monosaccharides, flavonoids, chalcones, and alcohols. Essential oils and organic extracts prepared from *V. tortilis* showed several biological properties, specifically antibacterial, antifungal, antiparasitic, antioxidant, antiproliferative, anti-diabetic, and anti-inflammatory effects. Antimicrobial and antiparasitic activities are due to the disturbance of cellular membranes and ultra-structural changes triggered by *V. tortilis* phytochemicals. While physiological and molecular processes such as apoptosis induction, preventing cell proliferation, and inflammatory mediators are responsible for the anti-diabetic, anti-cancer, and anti-inflammatory activities. However, further investigations concerning pharmacodynamics and pharmacokinetics should be carried out to validate their clinical applications.

## 1. Introduction

Fabaceae family contains several medicinal plants that have been recognized for their therapeutic potential in the treatment of a variety of ailments. Among these medicinal species, *V. tortilis* is a medicinal plant that is extensively employed in traditional pharmacopeia for its therapeutic properties [1]. This species distribution includes arid and semi-arid regions of northern, eastern, and southern Africa (Mauritania, Senegal, Mali, Niger, Chad and Sudan) and the Middle East (Palestine and the Arabian Peninsula) [2].

Biochemical and pharmacological studies have shown that *V. tortilis* exerts multiple biological properties, including antimicrobial, antidiabetic, anti-inflammatory and anticancer activities. Extracts and essential oils may, in fact, have antibacterial properties against a variety of bacteria, including multi-resistant strains (such as *Proteus mirabilis*, *Staphylococcus aureus*, *Pseudomonas aeruginosa*, and *Klebsiella pneumoniae*) to conventional antibiotics [3,4]. The antibacterial action of *V. tortilis* extracts has not been well addressed and appears to be linked to cell membrane disruption and structural changes. Furthermore, *V. tortilis* has been shown to have other antimicrobial properties against fungi such as *Candida albicans* and *Candida maltose* linked to human pathologies [5,6].

Oxidative stress produces free radicals, which are involved in the pathogenesis of complicated diseases such as chronic inflammation, diabetes, and cancer. *V. tortilis* extracts have shown an important capacity in the elimination of free radicals used in in vitro tests which supports their potential use in traditional therapies against these stress-related diseases [7].

In addition, *V. tortilis* extracts have been demonstrated to have anti-diabetic, anti-inflammatory, and antiproliferative properties in vitro and in vivo experiments. Although the mechanisms were not really completely understood, studies reveal that the plants’major components have cellular and molecular effects.

Indeed, *V. tortilis* extracts and essential oils are known for their high concentration of bioactive components such as flavonoids, chalcone and fatty acids with a remarkable variability depending on the region of collection, the parts used and the extraction methods used [4,8].

In this review, we explore studies performed on *V. tortilis* with respect to its taxonomy, medicinal properties, phytochemicals, and pharmacological properties. We will thus discuss the perspectives of applications of bioactive substances isolated from this plant.

## 2. Research Methodology

In this review, several scientific research databases were used to acquire data on the taxonomy, botanical description, ethnobotany, phytochemistry, and pharmacology of *V. tortilis*, including Google Scholar, Web of Science, Scopus, ScienceDirect, SpringerLink, Wiley Online, SciFinder, and PubMed. The obtained data were summarized according to each studied theme. Different keywords relating to *V. tortilis*, seed oil, extracts, biological effects, and chemical composition were used in this bibliometric survey. The reported phytochemical structures were established using ChemDraw Pro 8.0 and verified using the Pub Chemdatabase.

## 3. Results and Discussion

### 3.1. Taxonomy and Geographic Distribution

**Figure 1:**
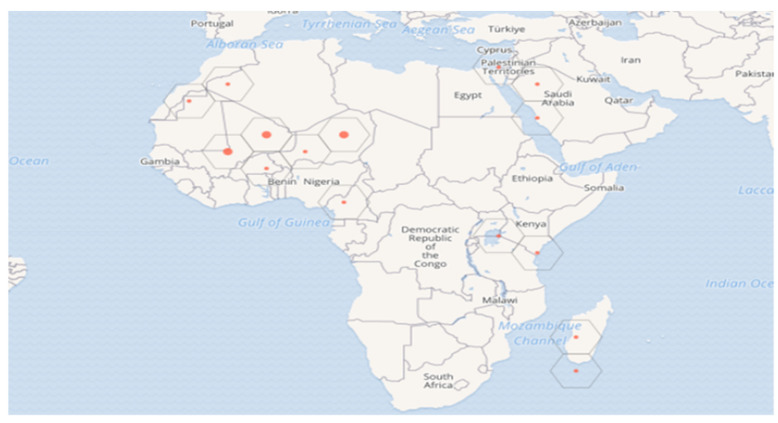
Geographic distribution of *V. tortilis.* The red dots in the figure legend illustrate the species intensity of distribution.

There are around 1200 species in the *Acacia* genus [9], endemic to the arid and semi-arid regions of northern and eastern Africa (Egypt, Libya, Tunisia, Algeria, and Morocco) and southern Africa (Mauritania, Senegal, Mali, Niger, Chad, and Sudan), as well as the Arabian Peninsula and the Middle East (Palestine; Figure 1) [2].

### 3.2. Botanical Description and Ecological Factors

In extremely dry conditions, a medium tree that is 4–15 m tall and has multiple trunks and an umbrella shape is reduced to a little wiry shrub (1 m tall maximum).

**Figure 2:**
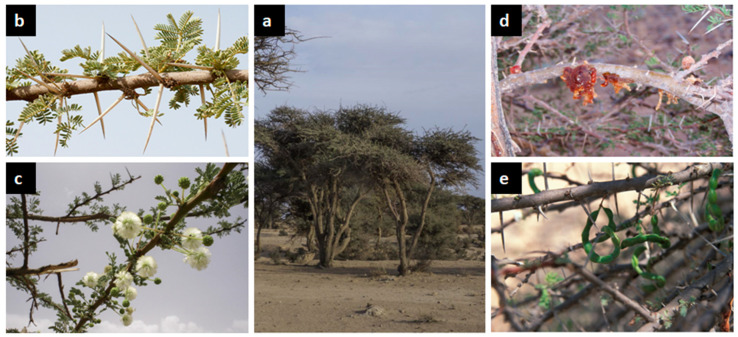
*V. tortilis* tree general aspect (**a**), leaves (**b**), flowers (**c**), gum (**d**) and pods (**e**) (https://www.teline.fr, accessed on 10 August 2022).

*V. tortilis* (Figure 2) bears little brownish hooked thorns and straight white thorns [10].

The immature stems and branches are reddish brown with grey lenticel stems, which transform to dark brown when mature, and the bark is a strong, fissured grey-brown-black color [11].

The pairs of spines are organized, with some spines being short and hooked (up to 5 mm long) and others being long and straight (up to 10 cm long). Having 4–10 pinnae and a maximum length of 2.5 cm, leaves can actually be large (15 leaflets pairs each). White, fragrant flowers are paired with flat, twisted, coil-like pods that resemble springtime. They blossom from May to June, bear fruit in July, and reach maturity from November to February. With longitudinal veins slightly constricted between the seeds, post-floral yellow-brown pods measuring 5–15 cm long and containing 5–18 seeds are present [9,10,11].

The *V. tortilis* tree can be found in a wide variety of environments, from subtropical deserts to tropical deserts to very dry forests. With an estimated minimum annual precipitation requirement of 10 mm and a maximum of over 100 mm, an expected annual temperature of 18–28 °C, and a pH of 6.5–8.5, it can withstand a wide range of precipitation. This species can withstand temperatures of up to 50 °C in hot, arid conditions. The umbrella tree thrives in the lowlands. It develops in regions where the annual rainfall exceeds 1000 mm. It is also drought tolerant, surviving in climates with an annual rainfall of less than 100 mm and a long and erratic dry season. The tree thrives in soils with a neutral pH. It develops lengthy roots in soils that are at least 0.25 m deep. The plant remains shade tolerant in shallow soil and must be spaced widely to allow for the growth and proliferation of its lateral roots [10].

### 3.3. Ethnomedicinal Use

*V. tortilis* is one of the most useful medicinal plants in Morocco, particularly in the southern area. Plant parts are employed in folk medicine for a variety of medicinal benefits. Different traditional uses of *A. tortilis* in different countries to treat different pathologies are shown in Table 1.

The pharmacological effects of the leaves of *V. tortilis* include antibacterial, antidiabetic, antiparasitic, cytotoxic, and anti-inflammatory characteristics [12,13,14]. In traditional medicine, root extracts are applied as cytotoxic and antiparasitic medicines [14]. Similarly, fruits exhibit cytotoxic and antimicrobial properties [15]. However, the gum serves as an antibacterial agent [3].

*V. tortilis* materials are prepared according to various methods to treat a variety of disorders (Table 1). Infusion, decoction and powdered materials are used in traditional medicinal preparations.

In Moroccan pharmacopeia, stomach disorders and diabetes were the most common medical applications of *V. tortilis* [1,12]. In fact, roots, fruits, and leaves were used as both decoctions and powders by the population of the Al Haouz Rhamna region to treat diabetes [12]. However, plant pod powder is used by the Moroccan Sahara population in Tan Tan [16].

Digestive problems are relieved with powdered leaves [17]. The bark of *V. tortilis* has been used in traditional medicine as an astringent, demulcent, detergent, hemostatic, and expectorant for angina, neuralgia, asthma, hepatitis, and jaundice [1]. Furthermore, the Tata province’s inhabitants (Morocco) used fruit to manage kidney stones using a decoction [1]. They are also used, by mixing the powder with olive oil as a poultice, for burns treatment [17].

In the Southern Algerian Sahara (Tassili N’ajjer), Hammiche and Maizahave reported that powdered fruit and seeds exert an effect against stomach diseases, diarrhea, and aches [19]. However, in Yemen, only *V. tortilis* fruits are used for digestive problems and similar purposes [15]. In addition, roots reduce bloating in the population of Samburu in Northern Kenya [18]. In Central Sudan (Soba area), *V. tortilis* is known to be used for malaria, swollen joints and skin disorders [20]. The local population of the Eastern Desert of Egypt (Wadi El-Gemal National Park) uses *V. tortilis* gum against jaundice, stomach acidity and ocular affections [21].

In Northern Burkina Faso, *V. tortilis* is used in traditional medicine for tropical infectious diseases such as schistosomiasis and malaria, as well as other illnesses (pulmonary and urogenital) [23]. Additionally, people in many parts of Africa use the trunk bark, which has diuretic and hypotensive qualities [13].

### 3.4. Phytochemical Compounds

The main compounds included in *V. tortilis* vegetable oil, essential oils, and extracts are listed in Table 2. The materials used, the type of extract, the chemical compounds found, and the corresponding chemical classes are all stated in this table for each nation.

According to the literature, chromatographic and spectroscopic techniques were used to identify the phytochemicals of *V. tortilis.* Chemical compositions reported in the literature are different due to the plant parts utilized and the countries from which they were procured (Table 2). The phytochemicals of *V. tortilis* are listed below according to the corresponding chemical classes.

#### 3.4.1. Fatty Acids

The chemical properties of *V. tortilis* seed oil showed a significant proportion of fatty acids. Linoleic acid was found to be the most abundant (381.3 ± 3.7–685.8 ± 5.3 g kg^−1^), oleic acid (190.0 ± 3.4–386.2 ± 4.06 g kg^−1^), has been followed by palmitic acid (74.2 ± 2.1–192.5 ± 1.5 g kg^−1^), stearic acid (16.8 ± 1.6–104.0 ± 6.6 g kg^−1^), and linolenic acid (7.9 ± 0.5–15.9 ± 0.6 g kg^−1^) [29].

Fatty acids are mainly extracted from seed oil with petroleum ether (40–60 °C) using a soxhlet. Linolenic acid was found to be the most abundant, then palmitic acid, oleic acid, and stearic acid [28]. However, in Egypt (South and North Sinai) fatty acid profile was different, and linoleic acid was the main component [8].

#### 3.4.2. Monosaccharides

The monosaccharide compounds were identified in the gum exudates of *A. tortilis*. The carbohydrate composition has only been documented through one research from India. It includes mostly L-arabinose, D-galactose, L-rhamnose, D-mannose, and D-glucose [26].

#### 3.4.3. Flavonoids and Chalcone

**Figure 3:**
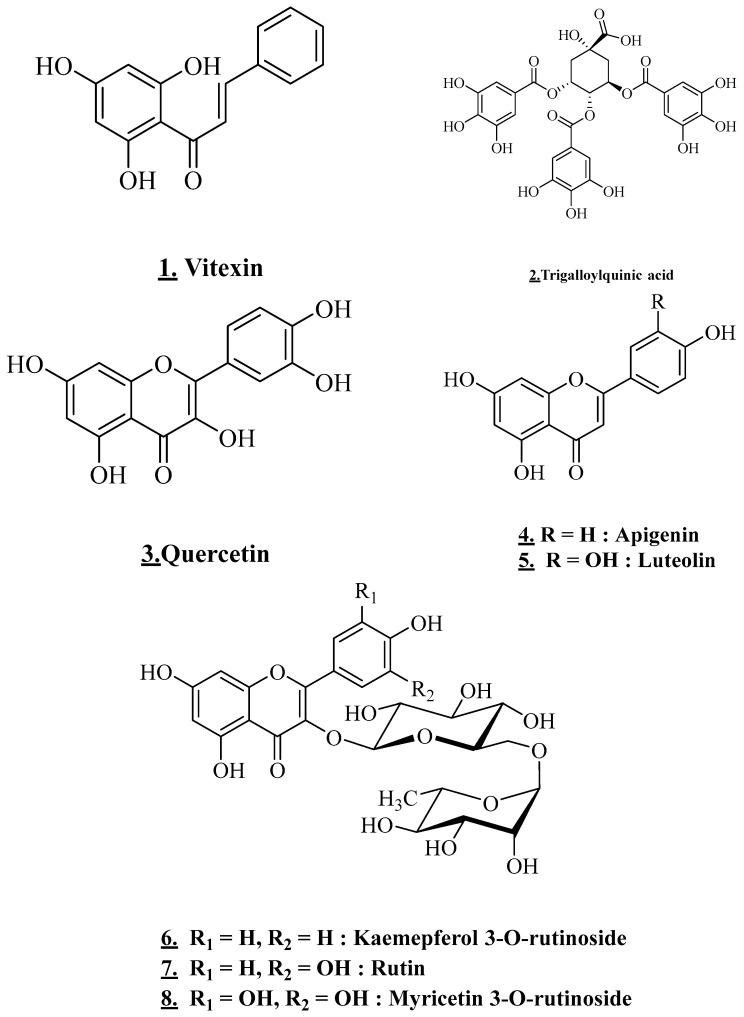
Structures of the main flavonoids, (**1**), (**2**), (**3**), (**4**), (**5**), (**6**), (**7**)**,** (**8**).

Flavonoids are a type of secondary metabolite found mostly in plant pigments and are responsible for flowers’ and fruits’ coloring. They could be used to treat a wide range of disorders. In *V. tortilis* preparations, the analytical analysis revealed a considerable group of flavonoid components (Table 2, Figure 3). The ethanolic extract of leaves collected in Algerian Sahara revealed the presence of flavonoids, including flavanol, epigallocatechin-3,7,3′,4′,5′-penta-Ogallate, epigallocatechin-3,5,4′,5′-tetra-Ogallate, (epi)gallocatechin-3,5′di-O-gallate, epigallocatechin-3,5,3′-tri-O-gallate, tri galloylquinic acid, (epi)gallocatechin-5,7-di-O-gallate, epigallocatechin-3,5,5′-tri-O-gallate, epigallocatechin-5,7,4′-tri-O-gallate, epigallocatechin-3,7,5′-tri-O-gallate, epigallocatechin-3,5,4′-tri-O-gallate [4].

The flavonol glycoside content of an ethanolic extract of *V. tortilis* leaves from Egypt is considerable. Myricetin 3-o-rutinoside, rutin (quercetin 3-o-rutinoside), kaemepferol 3-orutinoside were listed [24]. In addition, flavonoids such as isoflavone, flavones, and flavanols, 5,7-dihydroxy-4- p-methyl benzyl isoflavone, apigenin, luteolin and quercetin are mainly encountered in the methanolic extract of Yemen’s leaves [25].

**Figure 4:**
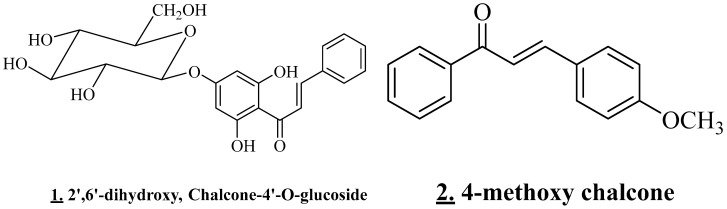
Structures of the main chalcone (**1**), (**2**).

Chalcone glycosides (Figure 4) were detected in the methanolic extract obtained by maceration. *V. tortilis* tested was collected from Alwadeha, Saudi Arabia. 2′,6′-dihydroxy, chalcone-4′-O-glucoside, and 4-methoxy chalcone were identified. Flavones, including vitexin, were also detected [25].

#### 3.4.4. Alcohols

**Figure 5:**
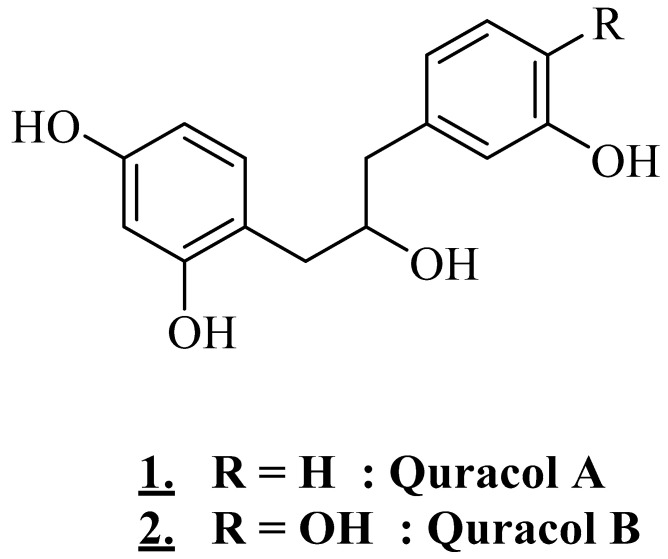
Structures of the main alcohols in *V. tortilis* (**1**), (**2**).

A study conducted on Acacia (Figure 5) collected in Somalia showed that an aqueous extract of stem bark covered with gum (decoction) contains alcohols. Uracol A [(2,4-dihydroxyphenyl)-3-(3-hydroxyphenyl)-propan-2-ol] and quracol B [1-(2,4-dihydroxphenyl)-3-(3,4-dihydroxyphenyl)- propan-2-ol] were also identified [27].

Plants have grown in popularity as functional food ingredients. Phytochemical profiles vary depending on the season, growth conditions, location, and environmental changes. The chemical composition of *V. tortilis* extracts differs qualitatively and quantitatively depending on the plant part used, the plants’ growing area, and the period of the vegetative phase.

### 3.5. Biological Properties

#### 3.5.1. Antibacterial Activity

Previous investigations reveal the antibacterial effects of this medicinal plant against Gram-positive and Gram-negative bacteria species. Table 3 summarizes antibacterial investigations conducted on extracts of various *V. tortilis* parts. Inhibition zone diameters (Ø) and minimum inhibitory and bactericidal concentrations (MIC and MBC) data are mentioned.

Ethanolic, chloroform, and acetonic extracts obtained from the aerial segment of *V. tortilis* antimicrobial properties of were studied against *Staphylococcus aureus (ATCC25923)* and *Pseudomonas aeruginosa* (ATCC 27853) in vitro by Abdllha and collaborators [5]. The strains were sensitive to ethanolic and acetonic extracts with diameter zones of inhibition ranging from 18 to 23 mm. Whereas no activity was found with the chloroform extract. The antibacterial property of extracts from the aerial section of *V. tortilis* was also investigated by the Alajmi team. The results revealed that ethanolic extract inhibited the proliferation of *Escherichia coli* (Ø = 19 ± 0.8 mm; MIC = 0.8 mg/mL), *Staphylococcus aureus* (Ø = 17 ± 0.9 mm; MIC = 0.4 mg/mL), and *Pseudomonas aeruginosa* germs (Ø = 16 ± 1.5 mm; MIC = 0.8 mg/mL) [30]. These differences in inhibition activities are certainly due to the difference in the plant parts used, the extraction technique, and edaphic factors. In another study, chloroform, alcoholic, petroleum ether, methanolic, and petroleum Benzin extracts of *V. tortilis* extracts, prepared from dry bark and fresh leaves were tested against *Klebsiella oxytoca*, *Staphylococcus aureus*, *Proteus mirabilis*, *Klebsiella pneumoniae*, and *Pseudomonas aeruginosa.* The diameter of the inhibition zone in this research ranged between 7–16 mm, with *Klebsiella pneumoniae* being the most sensitive bacteria, including all extracts [6]. Another study showed the antibacterial action of an aqueous extract generated from plant gum by using the disk diffusion method. *Salmonella typhi*, *Escherichia coli*, *Staphylococcus aureus*, and *Bacillus subtilis* have antibacterial activity with inhibition zone diameters of 19 ± 0.5, 17 ± 0.4, 24 ± 0.6, and 23 ± 0.1 mm, respectively [3].

Aqueous and ethanolic extracts produced from fresh leaves were tested for antibacterial activity using MIC and MBC against *Escherichia coli*, *Morganella morganii*, *Proteus mirabilis*, *Pseudomonas aeruginosa*, *Enterococcus faecalis*, *Klebsiella pneumoniae*, and *Listeria monocytogenes*. The MIC and MBC values varied from 1.25 to 5 mg/mL and 20 mg/mL, according to the results of [4].

Al-Fatimi and company investigated the antibacterial activity of dichloromethanic, methanolic, and aqueous extracts collected from the plant’s fruit using disc diffusion and minimum inhibitory concentrations methods. When compared to other extracts tested, dichloromethanic has a significant antimicrobial effect against *Staphylococcus aureus* (Ø = 20 mm; MIC = 500 µg/mL), *Bacillus subtilis* (Ø = 20 mm; MIC = 500 µg/mL), and *Micrococcus flavus* (Ø = 15 mm; MIC = 1000 µg/mL) [15].

Chloroform, petroleum ether, and methanol extracts inhibited more strains such as *Klebsiella pneumoniae*, *Klebsiella oxytoca*, and *Pseudomonas aeruginosa* compared to other types of extracts such as an ethanolic extract and an aqueous extract [6]. The results of the experiments with dried bark, which have higher inhibitory diameters than the experiments with leaves, were reversed [6]. Leaves inhibit more *Klebsiella pneumonia* than dry bark, later inhibits *Proteus mirabilis* more than *Staphylococcus aureus* [6]. The gum component inhibits Gram-positive bacteria, such as *Staphylococcus aureus* and *Bacillus subtilis*, as well as Gram-negative bacteria, including *Salmonella typhi* and *Escherichia coli* [3]. Gram-negative bacteria resistance to *V. tortilis* extracts is due to the enveloping outer membrane encircling the cell wall, which prevents hydrophobic chemicals from diffusing through the lipopolysaccharide. The main and synergistic effects of the major and minor chemical components, notably flavonoids (chalcone glycosides, flavones, flavanol, flavonol glycoside, isoflavone, flavone, and flavanols), monosaccharides, alcohols, and fatty acids, are frequently attributed to extracts antibacterial action(linolenic, linoleic, palmitic, oleic, stearic acids) [4,8,24,25,26,27,28,31].

Although plant extracts have been proven effective against these strains, the mode of action has yet to be discovered, necessitating more investigation into their mechanism of action.

#### 3.5.2. Antifungal Activity

*V. tortilis* antifungal activity was previously described in the literature against only three fungal strains [5,6,29]. Table 4 lists the data of previous studies on antifungal investigations from various parts of *V. tortilis* extracts.

Using the agar-plate well diffusion method, Abdllha and his associates tested the antifungal activity of aerial part extracts against *Candida albicans (ATCC90028)*. The acetonic extract (25 mm) and the ethanolic extract (23 mm) had the most activity against yeast, whereas the chloroform extract showed no inhibition [5].

Agar well diffusion method was also used [30] to determine the antifungal effect property of the aerial parts ethanolic extract against *Candida albicans*. Minimal inhibition concentration was also determined (0.8 mg/mL), which is of the same value as *Acacia salicina* (0.8 mg/mL), lower than *Acacia laeta* (1.6 mg/mL) and *Acacia hamulosa* (3.2 mg/mL).

Using the agar diffusion method, Al-Fatimi and colleagues found that the methanolic extract of fruit from *V. tortilis* had antifungal activity against *Candida maltosa*. The extract effectively suppressed proliferation, with an inhibition zone equal to 8 mm [15].

Ref. [6] and co-workers carried out the agar well diffusion method to evaluate the antifungal properties of methanolic extract, chloroform extract, petroleum benzin extract, and petroleum ether extract from *V. tortilis* aerial parts and dry bark against *Candida albicans* [6]. According to the experimental fungal growth, five extracts from the aerial parts exhibited inhibitory effects differently. As a result, the highest activity against *Candida albicans* was found using the methanolic and alcoholic extracts (Ø = 16.6 ± 0.67 and 16.6 ± 0.33 mm, respectively), subsequently the chloroform extract (Ø = 16.0 mm). The zones of inhibition for petroleum benzin and petroleum ether extracts, respectively, were 15.6 ± 0.33 mm and 15.3 ± 0.33 mm. Furthermore, similar *Candida albicans* inhibition activities were obtained using methanolic, petroleum benzin, and chloroform extracts from the dry bark of *V. tortilis* (Ø = 12.3 mm). Petroleum ether and alcohol exhibited a slight difference in this antifungal activity (Ø = 11.0 ± 1.52 and 10.3 ± 1.76 mm, respectively [6].

As previously stated, the differences in the results are related to genetic variability, environmental influences, and chemical composition. Thus, the antifungal activity is mainly due to the bioactive phytochemicals of *V. tortilis* (fatty acids, monosaccharides, flavonoids, and alcohols). Polyphenols are a significant class of natural compounds that includes flavonoids [32].

#### 3.5.3. Antiparasitic Effects

Malaria is a serious tropical disease caused by protozoa of the Plasmodium genus. The anti-plasmodial activity of *V. tortilis* chloroform extract against chloroquine (CQ)-resistant plasmodium was investigated in vitro (D6 clone).In comparison to chloroquine (IC_50_ = 0.004 g/mL), the results showed an IC_50_ equal to or higher than 10.0 µg/mL [14]. Another analysis revealed that methanolic extract exhibited anti-plasmodial potential in vitro, with an IC_50_ value of 85.73 ± 3.36 µg/mL [33]. The anti-leishmanial activity of aqueous and methanol extracts from the leaves of *V. tortilis* appears promising against *Leishmania major* (IDU/KE/83 NLB-144 strain) [33].

#### 3.5.4. Antioxidant Activity

Using all plant parts and processes, such as the DPPH, ABTS, FRAP, and carotene-linoleic acid approaches, several researchers have evaluated the antioxidant activity of various *V. tortilis* preparations (Table 5).

The antioxidant properties of *V. tortilis* ethanolic extract were measured by Alam and coworkers using the DPPH test [7]. The results were expressed as IC_50_ values, which were equal to 250.13 and 747.50 µg/mL, respectively.

According to the literature, at 500 µg/mL, the DPPH scavenging activity was in the following order: *A. salicina* > *A. laeta* > *A. tortilis* > *A. hamulosa* [7]. Surprisingly, the antioxidant activity of *Acacia salicina* was similar to that of the antioxidant standard rutin at amounts higher than 250 µg/mL. Acacia extracts’ activities were dose-dependent, according to the results of the DPPH radical scavenging assay.

In another work, a methanolic extract of *V. tortilis* leaves obtained by infusion and maceration exhibited considerable DPPH radical scavenging activity [23,35]. Results obtained were expressed as 84.3 ± 9.7 mg/g dry weight equivalent of chlorogenic acid.

The acetonic and ethanolic extracts of the aerial parts had the highest radical scavenging activity, with values of 82.04 and 83.02%, respectively [5]. However, the chloroform extract exhibits the lowest scavenging activity, with an RSA equal to 42.7%.

Using testing methods, including the ABTS test, Habib and colleagues demonstrated that aqueous extract by maceration of fruit *V. tortilis* exhibited an antiradical effect (80.62 ± 0.14%), by the DPPH antioxidant (19.12 ± 1.34%) [36]. According to the DPPH test, a methanolic extract from Acacia fruit maceration has a radical trapping activity of 26.17% (concentration = 100 µg/mL) in a study by Al-Fatimi and colleagues [15].

The antioxidant effect of maceration-based methanolic extract of Acacia seeds was studied using the DPPH and ABTS assays in another study. Data indicates that extract has the lowest activity, with EC_50_ values of 0.84 ± 0.03 and 2.22 ± 0.20 mM (TEAC), respectively [37].

Another investigation evaluated the antioxidant activity of *V. tortilis* gum aqueous extract obtained by maceration using the DPPH assay. As a result, 20 mg/mL gum extract exhibited a scavenging potential of 92.13 ± 0.13% [3].

The methods conducted to evaluate antioxidant activity have a specific mechanism, which explains the diversity in the results listed in Table 5. These approaches are, in effect, mutually beneficial. Thus, in order to confirm and validate the results presented in the table of extracts from *A. tortilis*, in vivo antioxidant activity tests should be performed.

#### 3.5.5. Antiproliferative Activity

Different analytical methods highlighted the cytotoxic activity of *V. tortilis,* as shown in Table 6. *V. tortilis* leaves revealed potential in vitro cytotoxic effect, according to Ziani and colleagues. The sulforhodamine B colorimetric assay was used to determine the capacity of extracts to inhibit cell proliferation in four human tumor cell lines: NCI–H460 (non-small cell lung cancer), HeLa (cervical carcinoma), HepG2 (hepatocellular carcinoma), and MCF-7 (breast carcinoma). Elipticine was used as a positive control, and the results were expressed as Growth Inhibition values (GI_50_, µg/mL). The growth of non-tumor liver cells (PLP2) was indeed tested using different amounts of *V. tortilis* extracts under the same conditions as tumor cell lines. Ethanolic extract expressed a low GI_50_ on the four tumor cell lines (from 33.3 to 53.0 µg/mL) [4]. Compared to the cytotoxicity data obtained by the ethanolic extract of aerial parts [30], ethanolic leaf extracts seem to have a high cytotoxic effect on HepG2 cells (IC_50_ 42.3 and 33 µg/mL, respectively).

Acacia species have been demonstrated to have a cytotoxic effect on a variety of tumor cell lines (HepG2, MCF-7, HEK-293) ([38]. A high GI_50_ (259 ± 0.05 μg/mL) was recorded on the PLP2 cell line using the ethanolic leaves extract [4]. The lower cytotoxicity of *V. tortilis* extracts in the normal PLP2 cell line might be due to the specific molecular mechanisms involved in tumor cells that are not present or active in normal cell lines.

Furthermore, tumor cells that were exposed to *V. tortilis* extracts had a higher response than non-tumor cells, implying that they were more reactive to the phenolic chemicals present in the extracts. These substances tend to be interfering with the cell cycle, apoptosis, and cell death pathways, all of which are implicated in tumor proliferation [39,40]. According to [41], the cytotoxic effect could be attributed to epigallocatechin derivatives, which are prevalent in *V. tortilis* extracts, as well as a possible synergistic interaction with different *Acacia* active components that approach epigallocatechin derivatives. As previously reported by [42], *V. tortilis* contains a significant amount of esterified gallic acid and epicatechin galloyled with a significant level of hydroxyl ring substitutions, which have also been shown to inhibit the proliferation of many tumor cell lines directly. This provides a coherent explanation for cytotoxicity on human tumor cells [4].

#### 3.5.6. Antidiabetic Effect

Investigations conducted on *V. tortilis’* anti-diabetic properties are limited. Table 7 reports the in vivo research data realized with different Acacia extracts.

Because diabetes is always related to body weight loss due to muscle wasting and tissue protein catabolism, the effect of the administration of AEATP on body weight was followed [46,47]. Indeed, AEATP markedly increased body weight on the 14th day of administration and balanced the effect of STZ approximately close to the glimepiride-treated group.

Kumar and Singh assessed the anti-diabetic activity of aqueous extract of *V. tortilis* polysaccharide from gum exudates on streptozotocin-nicotinamide-induced diabetic rats. Dosages of 250, 500, and 1000 mg/kg were administered to male albino Wistar rats for 28 days, with all necessary controls. AEATP reduces current weight, fasting blood glucose, total cholesterol, triglyceride, LDL, VLDL, SGOT, and SGPT levels while improving HDL levels [48].

Treatments were completed on the 21st and 28th days, and body weight was almost the same, and statistically, no significant difference was found between the glimepiride and polysaccharide tests [48]. This could be ascribed to the protective effect of polysaccharides in the control of muscle wasting, contrary to neoglucogenesis, as well as increased insulin secretion and enhanced glycemic control.

After administering polysaccharide, glycated hemoglobin was also substantially reduced (250–1000 mg/kg) as compared to the STZ-induced diabetic group with close value to those of glimepiride after 28 days of treatment [48]. Flavonoids abundant in *V. tortilis* are known for their ability to attenuate hyperglycemia and reduce the nonenzymatic glycation of proteins in animals [25,49,50]. Nutritional flavonoids or extracts rich in flavonoids may prevent and treat T2DM, as well as reduce diabetes complications, according to significant variables from cell and animal models. Flavonoids in the diet lower blood glucose levels by protecting pancreatic cells, stimulating insulin signaling, inducing the pancreas to release insulin, inhibiting glycogenolysis, and inhibiting neoglucogenesis, digestive enzymes, and carbohydrate metabolizing enzymes, as well as inhibiting neoglucogenesis, digestion enzymes, and carbohydrate metabolizing enzymes [51].

Certain flavonoids aglycones and their O-glycosides/C-glycosides exhibited anti-diabetic effects in animal models, namely apigenin, baicalein, quercetin, kaempferol, myricetin, daidzein, luteolin, and naringenin. Leading to a shortage of data and varied animal models, determining the structure-activity interaction of flavonoids and antidiabetic effects remains difficult [51].

**Figure 6:**
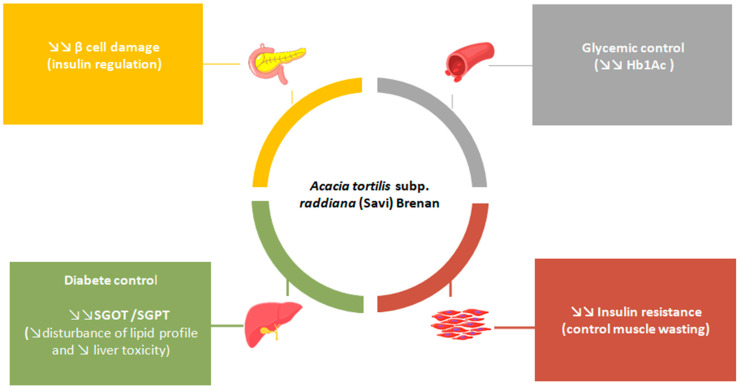
In vivo antidiabetic mechanism effects of *V. tortilis*.

Lipids are well-known for their role in the pathophysiology of diabetic complications and the resulting hyperlipidemia. Diabetics have a higher risk of developing atherosclerosis than non-diabetics [52]. Interestingly, polysaccharides have shown a considerable reduction in TG and LDL-C, implying additional hypolipidemic activity that could protect against the development of cardiovascular disease and diabetes. Cholesterol levels in serum significantly decreased from 206.1 ± 3.7 mg/dL (STZ-induced diabetic group), and both 500 and 1000 mg/kg of polysaccharide normalized the elevated cholesterol to levels similar to that of glimepiride (134.2 ± 3.52 mg/dL) [48]. In the same study, Bhateja and Singh also showed that levels of total triglyceride (TG), low-density lipoprotein (LDL), and very low-density lipoprotein (VLDL) levels were significantly reduced after administration (Figure 6). The same effectiveness was reported for VLDL levels. Otherwise, HDL levels were significantly reduced in the diabetic control group, which was close to that of glimepiride (24.44 ± 1.3 mg/kg) [48].

Regarding the effect of polysaccharides on liver enzymes, high levels of SGOT (284.5 ± 10.21) and SGPT (161.9 ± 5.21 unit/L) in the serum of diabetic rats were lowered after administration of AEATP [48]. Normalized serum SGPT and SGOT levels are responsible for normal liver function in reversing diabetes-related organ damage.

Decreased fasting insulin levels (0.373 ± 0.026 ng/mL) and the insulin content of the pancreas (56.0 ± 2.81 ng/mg pancreas) in STZ-induced diabetic rats were also improved by AEATP [48]. Polysaccharides may improve insulin secretion by membrane depolarization, inhibiting K^+^-ATP channels and stimulating Ca^2+^ influx. Since altered content of gut incretin is associated with type 2 diabetes in animal models, polysaccharides may stimulate incretin secretion and thus probably induce glucagon-like peptide-1 (GLP-1) increase or dipeptidyl peptidase-4 (DPP-4) inhibition [53]. Moreover, it shows that the polysaccharide effect, similar to STZ treatment, may include a reduction in oxidative stress and, consequently, oxidative damage and dyslipidemia prevention.

#### 3.5.7. Anti-Inflammatory Effect

A murine macrophage-like cell line was used to study the anti-inflammatory properties of ethanolic and aqueous extracts of *V. tortilis* leaves collected in the Algerian Sahara [4]. RAW 264.7 cell model was stimulated by lipopolysaccharide (LPS). The anti-inflammatory activities could be explained by the presence of phenolic compounds, particularly (epi)-gallocatechin derivatives. Phenolic compounds have been shown to inhibit inflammatory responses in activated Raw 264.7 cells through NO production reduction as well as inhibition of proinflammatory mediators and cytokines, including TNF-α, IL-1, IL-6, and IL-12. The use of (epi)-catechin to reduce NO production in the studied cell model has been previously reported in the literature [54,55]. In addition, a gallate ester substitution is involved in the anti-inflammatory properties of (epi)-catechins and gallic acid enhancement [56]. Epigallocatechin-3-gallate is an efficient scavenger ROS and RNS such as NO and peroxynitrite involved in the nucleus translocation of NF-kB nuclear factor from the cytoplasm [57]. Moreover, it has been previously reported that the suppression of iNOS and COX-2 enzymes and TNF-α expression is due to the reduction of NO production by acetone extract of *Acacia* stem bark [58].

In the study conducted by [24], ethanolic extract of *V. tortilis* leaves exhibited significant inhibition of COX-1 and COX-2. These enzymes are often targeted in the inflammatory response [59]. The observed effect is certainly ascribed to the richness of *V. tortilis* extract with rutin and catechin.

## 4. Conclusions and Perspectives

This review covered the taxonomy, medical applications, phytochemistry, and pharmacological characteristics of *V. tortilis*. According to published data, the biological activities of *V. tortilis,* as supported by conventional medical uses, are implicated by bioactive substances of plant sections cited (fruit, seeds, gum, leaves, and roots). These phytochemicals, which are involved in biological processes, include fatty acids, flavonoids, and chalcones. The fact that other compounds have not yet been recognized and classified as terpenoids makes a significant recommendation for additional phytochemical research.

Additionally, *V. tortilis* extracts show exceptional pharmacological qualities, such as anti-inflammatory, anti-microbial, antioxidant, and anti-diabetic action. These characteristics include cellular and molecular activities on many pathology-causing sites. Researched works revealed that the primary bioactive components of *V. tortilis* are the mediators of these pathways.

To confirm their effectiveness, additional research into the pharmacodynamic activities of these key bioactive chemicals needs to be done. Additionally, studies on the pharmacokinetics of the primary bioactive chemicals in *V. tortilis* should be conducted to demonstrate their safety.

This study provides a conventional analysis using pharmacological justifications on the functional advantages of the various *V. tortilis* plant parts that could be used, on the one hand, in in vivo studies for the creation and innovation of therapeutic strategies to prevent and manage type 2 diabetes, and, on the other hand, in the pharmaceutical industry, use of nanoparticles and the formulation based on non-toxic natural resources.

## Figures and Tables

**Table 1:** Traditional uses of different parts of *V. tortilis*.

Study of Area	Used Part	Mode of Preparation	Traditional Use	References
Morocco (Al Haouz Rhamna region)	Root, fruit and leaf	Decoction and powder	Diabetes	[12]
Moroccan Sahara (Tan Tan)	Plant pod	Powder	Diabetes	[16]
Morocco (Tata Province)	Gum	Infusion	Neuralgia, asthma, hepatitis, jaundice	[1]
	Bark	Infusion	Astringent, demulcent, haemostatic, expectorant, angina	
	Fruit	Decoction	Kidney stones	
Morocco (Agadir Ida Outanane)	Leaves	Powder	Diarrhea, stomach diseases, burns	[17]
	Leaves	Poutine of powder mixed with olive oil		
Northern Kenya (Samburu)	Roots	Not reported	Minimize bloating	[18]
Southern Algerian Sahara (Tassili N’ajjer)	Fruit	Powder	Stomach diseases, diarrhoeaaches	[19]
Central Sudan (Soba area, Khartoum State)	No reported	Not reported	Malaria, swollen joint problems, skin allergies	[20]
Eastern Desert of Egypt (Wadi El-Gemal National Park)	Gum	Not reported	Stomach acidity, ocular affections, jaundice	[21]
Yemen	Fruits	Not reported	Stomach aches, digestive disorders	[15]
Some regions of Africa	Leaves, trunk bark	Not reported	Jaundice, bilious fevers, skin allergies, diabetes, hypertension, diuretic properties	[13]
Northern Burkina Fasso	No reported	Not reported	Urogenital and pulmonary Infectious, schistosomiasis, ulcers, malaria, yellow fever, dysentery	[22]

**Table 2:** Chemical composition of different *V. tortilis* plant parts extracts.

Part Used	Origin	Type of Extract/ Seed Oil	Chemical Composition	Compounds Class	References
Leaves	Saudi Arabia	Methanolic Extract	2′,6′-dihydroxy, Chalcone-4′-O-glucoside, 4-methoxy chalcone	Flavonoids (chalcone glycosides)	[11]
			Vitexin	Flavonoids (flavones)	
Leaves	Algerian Sahara	Ethanolic extract	Epigallocatechin-3,7,3′,4′,5′-penta-Ogallate;	Flavonoids (flavanol)	[4]
			Epigallocatechin-3,5,4′,5′-tetra-Ogallate;		
			(Epi)gallocatechin-3,5′di-O-gallate;		
			Epigallocatechin-3,5,3′-tri-O-gallate;		
			Trigalloylquinic acid;		
			(Epi)gallocatechin-5,7-di-O-gallate;		
			Epigallocatechin-3,5,5′-tri-O-gallate;		
			Epigallocatechin-5,7,4′-tri-O-gallate;		
			Epigallocatechin-3,7,5′-tri-O-gallate;		
			Epigallocatechin-3,5,4′-tri-O-gallate.		
Leaves	Egypt	Ethanol extract	Myricetin 3-O-rutinoside; Rutin (Quercetin 3-O-rutinoside); Kaemepferol 3-O-rutinoside	Flavonol glycoside	[24]
Leaves	Yemen	Methanol extract	5,7-dihydroxy-4-p-methyl benzyl isoflavone	Flavonoids (isoflavone)	[25]
			Apigenin Luteolin	Flavonoids (flavone)	
			Quercetin	Flavonoids (flavanols)	
Gum exudates	India	Aqueous extract	L-arabinose, D-galactose, L-rhamnose, D-mannose, D-glucose, D-galacturonic acid, and D-glucuronic acid	Monosaccharides and derivatives	[26]
Stem bark (with gum)	Somalia	Aqueous extract	Quracol A and Quracol B	Alcohols	[27]
Seeds	Israel	Seed oil	Linolenic acid, linoleic acid, palmitic acid, oleic acid, and stearic acid	Fatty acids	[28]
	Egypt		Linoleic acid, palmitic acid, stearic acid, oleic acid, and arachidic acids	Fatty acids	[8]

**Table 3:** Antibacterial activity of *V. tortilis*.

Use Part	Extract	Bacterial Strain	Key Results	References
Aerial part	Ethanolic extract	*Staphylococcus aureus (ATCC25923)*	Ø = 20 mm Control not reported	[5]
		*Pseudomonas aeruginosa (ATCC27853)*	Ø = 20 mm Control not reported	
	Chloroform extract	*Staphylococcus aureus* *(ATCC25923)*	nd	
		*Pseudomonas aeruginosa (ATCC 27853)*	nd	
	Acetonic extract	*Staphylococcus aureus (ATCC 25923)*	Ø = 23 mm Control not reported	
		*Pseudomonas aeruginosa (ATCC 27853)*	Ø = 18 mm Control not reported	
Aerial part	Ethanolic extract	*Staphylococcus aureus*	Ø = 17 ± 0.9 mm MIC = 0.4 mg/mL Ampicillin Ø = 21 ± 1.9 mm	[30]
		*Escherichia coli*	Ø = 19 ± 0.8 mm MIC = 0.8 mg/mL Doxycycline Ø = 25 ± 1.2 mm	
		*Pseudomonas aeruginosa*	Ø = 16 ± 1.5 mm MIC = 0.8 mg/mL Doxycycline Ø = 24 ± 1.7 mm	
Fruit	Dichloromethanic extract	*Staphylococcus aureus ATCC 29213*	Ø = 20 mm Ampicillin Ø = 26 mm	[15]
		*Bacillus subtilis ATCC 6059*	Ø = 20 mm Ampicillin Ø = 28 mm	
		*Micrococcus flavus SBUG*	Ø = 15 mm Ampicillin Ø = 31 mm	
	Methanolic extract	*Staphylococcus aureus ATCC 29213*	Ø = 10 mm Ampicillin Ø = 26 mm	
		*Bacillus subtilis ATCC 6059*	Ø = 8 mm Ampicillin Ø = 28 mm	
		*Micrococcus flavus SBUG*	Ø = 8 mm Ampicillin Ø = 31 mm	
		*Pseudomonas aeruginosa ATCC 27853*	Ø = 10 mm Gentamicin Ø = 18 mm	
	Aqueous extract	*Staphylococcusaureus ATCC 29213*	Ø = 10 mm Ampicillin Ø = 26 mm	
		*Micrococcus flavus SBUG*	Ø = 8 mm Ampicillin Ø = 31mm	
		*Pseudomonas aeruginosa ATCC 27853*	Ø = 8 mm Gentamicin Ø = 18 mm	
	Dichloromethanic extract	*Staphylococcus aureus ATCC 29213*	MIC = 500 µg/mL Ampicillin =0.05 µg/mL	
		*Bacillus subtilis ATCC 6059*	MIC = 500 µg/mL Control not tested	
		*Micrococcus flavus SBUG*	MIC = 1000 µg/mL Ampicillin = 0.25 µg/mL	
	Methanolic extract	*Staphylococcus aureus ATCC 29213*	MIC = 500 µg/mL Ampicillin = 0.05 µg/mL	
		*Pseudomonas aeruginosa ATCC 27853*	MIC = 1000 µg/mL Control not tested	
	Aqueous extract	*Staphylococcus aureus ATCC 29213*	MIC = 1000 µg/mL Ampicillin = 0.05 µg/mL	
Gum	Aqueous extract	*Salmonella typhi*	Ø = 19 ± 0.5 mm Control not tested	[3]
		*Escherichia coli*	Ø = 17 ± 0.4 mm Control not tested	
		*Staphylococcus aureus*	Ø = 24 ± 0.6 mm Control not tested	
		*Bacillus subtilis*	Ø = 23 ± 0.1 mm Control not tested	
Fresh leaves	Chloroform extract	*Klebsiella oxytoca*	Ø = 10.0 ± 0.57 mm Control not reported	[6]
		*Staphylococcus aureus*	Ø = 10.6 ± 0.66 mm Control not reported	
		*Proteus mirabilis*	Ø = 10.0 ± 0.57 mm Control not reported	
		*Klebsiella pneumoniae*	Ø = 14.3 ± 0.88 mm Control not reported	
		*Pseudomonas aeruginosa*	Ø = 9.3 ± 0.33 mm Control not reported	
	Alcoholic extract	*Klebsiella oxytoca*	Ø = 12.0 ± 1.15 mm Control not reported	
		*Staphylococcus aureus*	Ø = 9.3 ± 0.33 mm Control not reported	
		*Proteus mirabilis*	Ø = 8.6 ± 0.66 mm Control not reported	
		*Klebsiella pneumoniae*	Ø = 15.0 ± 0.57 mm Control not reported	
		*Pseudomonas aeruginosa*	Ø = 11.0 ± 0.00 mm Control not reported	
	Petroleum ether extract	*Klebsiella oxytoca*	Ø = 10.6 ± 0.66 mm	
		*Staphylococcus aureus*	Ø = 9.0 ± 1.00 mm	
		*Proteus mirabilis*	Ø = 8.3 ± 0.88 mm	
		*Klebsiella pneumoniae*	Ø = 14.3 ± 0.33 mm	
		*Pseudomonas aeruginosa*	Ø = 8.6 ± 0.33 mm	
			Control not reoprted	
	Methanolic extract	*Klebsiella oxytoca*	Ø = 13.0 ± 0.58 mm	
		*Staphylococcus aureus*	Ø = 11.3 ± 0.33 mm	
		*Proteus mirabilis*	Ø = 9.6 ± 1.30 mm	
		*Klebsiella pneumoniae*	Ø = 15.3 ± 0.33 mm	
		*Pseudomonas aeruginosa*	Ø = 11.3 ± 0.33mm	
			Control not reoprted	
	Petroleum Benzin extract	*Klebsiella oxytoca*	Ø = 9.3 ± 0.88 mm	
		*Staphylococcus aureus*	Ø = 8.6 ± 0.33 mm	
		*Proteus mirabilis*	Ø = 9.6 ± 1.45 mm	
			Control not reoprted	
		*Klebsiella pneumoniae*	Ø = 16.0 ± 0.00 mm	
		*Pseudomonas aeruginosa*	Ø = 8.6 ± 0.88 mm	
			Control not reoprted	
Dry Bark	Chloroform extract	*Klebsiella oxytoca*	Ø = 7.3 ± 0.33 mm	[6]
		*Staphylococcus aureus*	Ø = 7.6 ± 0.66 mm	
		*Proteus mirabilis*	Ø = 12.0 ± 0.00 mm	
		*Klebsiella pneumoniae*	Ø = 11.3 ± 0.33 mm	
		*Pseudomonas aeruginosa*	Ø = 7.0 ± 0.00 mm	
			Control not reported	
	Alcoholic extract	*Klebsiella oxytoca*	Ø = 9.0 ± 0.57 mm	
		*Staphylococcus aureus*	Ø = 7.3 ± 0.33 mm	
		*Proteus mirabilis*	Ø = 13.3 ± 0.33 mm	
		*Klebsiella pneumoniae*	Ø = 13.6 ± 0.88 mm	
		*Pseudomonas aeruginosa*	Ø = 7.0 ± 0.00 mm	
			Control not reported	
	Petroleum ether extract	*Klebsiella oxytoca* *Staphylococcus aureus* *Proteus mirabilis* *Klebsiella pneumoniae* *Pseudomonas aeruginosa*	Ø = 7.6 ± 0.33 mm Ø = 8.3 ± 0.88 mm Ø = 12.3 ± 0.33 mm Ø = 12.3 ± 0.88 mm Ø = 7.6 ± 0.33 mm	
	Methanolic extract	*Klebsiella oxytoca* *Staphylococcus aureus* *Proteus mirabilis* *Klebsiella pneumoniae* *Pseudomonas aeruginosa*	Ø = 7.6 ± 0.67 mm Ø = 7.0 ± 0.00 mm Ø = 11.3 ± 0.88 mm Ø = 13.0 ± 0.58 mm Ø = 7.0 ± 0.00 mm	
	Petroleum benzin extract	*Klebsiella oxytoca* *Staphylococcus aureus* *Proteus mirabilis* *Klebsiella pneumoniae* *Pseudomonas aeruginosa*	Ø = 7.0 ± 0.00 mm Ø = 8.6 ± 0.67 mm Ø = 12.0 ± 0.00 mm Ø = 13.3 ± 0.33 mm Ø = 7.3 ± 0.33 mm	
Fresh leaves	Aqueous extract	*Escherichia coli*	MIC = 1.25 mg/mL; MBC = 20 mg/mL Ampicillin (20 mg/mL) < 0.15	[4]
		*Klebsiella pneumoniae*	MIC = 2.5 mg/mL Ampicillin (20 mg/mL) = 10 MBC = 20 mg/mL Ampicillin (20 mg/mL) = 20	
		*Morganella morganii*	MIC = 1.25 mg/mL Ampicillin (20 mg/mL) = 20 MBC = 20 mg/mL Ampicillin (20 mg/mL) > 20	
		*Proteus mirabilis*	MIC =2.5 mg/mL ; MBC = 20 mg/mL Ampicillin (20 mg/mL) < 0.15	
		*Pseudomonas aeruginosa*	MIC = 2.5 mg/mL ; MBC = 20 mg/mL Ampicillin (20 mg/mL) > 20	
		*Enterococcus faecalis*	MIC = 5 mg/mL ; MBC = 20 mg/mL Ampicillin (20 mg/mL) < 0.15	
		*Listeria monocytogenes*	MIC = 1.25 mg/mL ; MBC = 20 mg/mL Ampicillin (20 mg/mL) < 0.15	
Fresh leaves	Ethanolic extract	*Escherichia coli*	MIC = 1.25 mg/mL; MBC = 20 mg/mL Ampicillin (20 mg/mL) < 0.15	
		*Klebsiella pneumoniae*	MIC = 1.25 mg/mL Ampicillin (20 mg/mL) = 10 MBC = 20 mg/mL Ampicillin (20 mg/mL) = 20	
		*Morganella morganii*	MIC = 1.25 mg/mL Ampicillin (20 mg/mL) = 20 MBC = 20 mg/mL Ampicillin (20 mg/mL) > 20	
		*Proteus mirabilis*	MIC =1.25 mg/mL; MBC = 20 mg/mL Ampicillin (20 mg/mL) < 0.15	
		*Pseudomonas aeruginosa*	MIC = 1.25 mg/mL; MBC = 20 mg/mL Ampicillin (20 mg/mL) > 20	
		*Enterococcus faecalis*	MIC = 2.5 mg/mL; MBC = 20 mg/mL Ampicillin (20 mg/mL) < 0.15	
		*Listeria monocytogenes*	MIC = 1.25 mg/mL; MBC = 20 mg/mL Ampicillin (20 mg/mL) < 0.15	

**Table 4:** Antifungal activity of *V. tortilis* (none of the references cited below reported control data).

Use Part	Type of Extract	Tested Microorganisms	Key Results	References
Aerial part	Ethanolic extract	*Candida albicans (ATCC90028)*	Ø = 23 mm	[5]
	Chloroform extract		nd	
	Acetonic extract		Ø = 25 mm	
Aerial part	Ethanolic extract	*Candida albicans*	Ø = 15 ± 1.0 mm	[30]
			MIC = 0.8 mg/mL	
Fruit	Methanolic extract	*Candida maltose*	Ø = 8 mm	[15]
Aerial part	Chloroform extract	*Candida albicans*	Ø = 16.0 ± 0.00 mm	[6]
	Alcoholic extract		Ø = 16.6 ± 0.33 mm	
	Petroleum ether extract		Ø = 15.3 ± 0.33 mm	
	Methanolic extract		Ø = 16.6 ± 0.67 mm	
	Petroleum benzin extract		Ø = 15.6 ± 0.33 mm	
Dry Bark	Chloroform extract	*Candida albicans*	Ø = 12.3 ± 1.20 mm	[6]
	Alcoholic extract		Ø = 10.3 ± 1.76 mm	
	Petroleum ether extract		Ø = 11.0 ± 1.52 mm	
	Methanolic extract		Ø = 12.3 ± 0.33 mm	
	Petroleum benzin extract		Ø = 12.3 ± 0.33 mm	

**Table 5:** Antioxidant activity of *V. tortilis*.

Use Part	Extracts/Method Extraction	Used Method	Key Results	References
Aerial parts	Ethanolic extract/maceration	DPPH	RSA = 83 ± 0.02% Control not reported	[5]
	Chloroform extract/maceration	DPPH	RSA = 42 ± 0.7% Control not reported	
	Acetonic extract/maceration	DPPH	RSA = 82 ± 0.04% Control not reported	
Leaves	Ethanolic extract/ultrasound	DPPH	IC_50_ = 250.13 µg/mL Rutin IC_50_ = 250.13 µg/mL	[7]
Leaves	n-Butanol extract/maceration	DPPH	RSA = 89.8% Control not tested	[34]
Leaves	Methanolic extract/infusion	DPPH	84.3 ± 9.7 mg/g DW (Chlorogenic acid equivalent) Control not tested	[35]
Leaves	Methanolic extract/maceration	DPPH	IC_50_ = 0.03 ± 0.01 µg/mL Trolox = 0.01 ± 0.00 µg/mL	[23].
Trunk bark	Methanolic extract/maceration	DPPH	IC_50_ = 0.01 ± 0.01 µg/mL Trolox = 0.01 ± 0.00 µg/mL	[23].
Fruit	Methanolic extract/maceration	DPPH	RSA = 26.17% (at 100 µg/mL) Ascorbic acid RSA = 96.9% (at 100 µg/mL)	[15]
Fruit	Aqueous extract/maceration	ABTS	RSA = 80.62 ± 0.14% Control not tested	[36]
		DPPH	RSA = 19.12 ± 1.34% Control not tested	
Seeds	Methanolic extract/maceration	DPPH	RSA = 0.84 ± 0.03 (TEAC mM) Control not tested	[37]
		ABTS	RSA = 2.22 ± 0.20 (TEAC mM) Control not tested	
Gum	Aqueous extract/maceration	DPPH	RSA = 92.13 ± 0.13% (at 20 mg/mL) Control not tested	[3]

**Table 6:** Antiproliferative activity of *V. tortilis*.

Activities	Use Part	Extracts	Experimental Approach	Key Results	References
Cytotoxic activity	Aerial part	Ethanolic extract	HepG2, HEK-293, MCF-7, and MDA-MB-231 cancer cells were tested in vitro for anticancer efficacy.	The estimated IC_50_ (μg·mL^−1^ ± SD): HepG2 (Liver) = 42.3 ± 1.78 5-Flurourasil = 3.1 ± 0.07 HEK-293 (Kidney) = 49.1 ± 1.92 5-Flurourasil = 2.5 ± 0.05 MCF-7 (Breast) = 65.7 ± 2.49 5-Flurourasil = 3.7 ± 0.07 MDA-MB-231 (Breast) = 52.2 ± 1. 995-Flurourasil = 3.9 ± 0.09	[30]
Cytotoxic activity	Fruit	Methanolic extract	FL-cells, a human amniotic epithelial cell line	IC_50_% (µg/mL) against FL-cells > 1000	[15]
Cytotoxic activity	Root bark	Chloroform extract	One human cancer cell line was used to test cytotoxic activity (KB, a human oral epidermoid cancer cell line)	Cytotoxicity assay; KB IC_50_ (µg/mL) > 20 Chloroquine = 17.4 µg/mL	[14]
Cytotoxic activity	Leaves	Ethanolic extract	Cytotoxicity, Growth inhibition values (GI50, μg/mL)	Cell lung cancer (NCI–H460) = 52 ± 1. Ellipticine = 1.0 ± 0.1 Cervical carcinoma (HeLa) = 48.2 ± 0.1. Ellipticine = 1.9 ± 0.1 Hepatocellular carcinoma (HepG2) = 33 ± 1 (*p* < 0.05) Ellipticine = 1.1 ± 0.2 Breast carcinoma (MCF-7) = 52 ± 1 (*p* < 0.05). Ellipticine = 0.91 ± 0.04 PLP2 = 259 ± 0.1 Ellipticine = 3.2 ± 0.7	[4]

**Table 7:** In vivo antidiabetic effects of *V. tortilis*.

Part Used	Extract Tested	Dose	Model	Keys Results	References
Seed	Aqueous extract	100 and 200 mg/kg body weight	Normoglycaemic and Alloxan-induced diabetic rats	Decreases blood glucose levels, fluid intake by 34.49%, and food intake	[43]
Leaves	Aqueous extract	800 mg/kg	Normoglycaemic rats	Reduces blood glucose, serum total cholesterol and LDL level, and body weight Increase serum HDL-cholesterol	[44]
Stem and branches	Aqueous extract	250–1000 mg/kg	Streptozotocin-Nicotinamide Induced diabetic rats	Minimizes fasting blood glucose level, glycated hemoglobin level, total cholesterol, triglyceride, LDL, VLDL, SGOT, and SGPT levels, and improved HDL level	[45]

## Abbreviations

**ABTS**	2,2’-Azino-bis (3-ethylBenzoThiazoline-6-Sulphonic)acid
**ATCC**	American Type Culture Collection
**COX-2**	Cyclooxygenase-2
**DPPH**	2,2-Diphenyl-1-picryl hydrazyl radical
**DW**	Dry Weight
**EC_50_**	Effective Concentration of 50%
**FRAP**	Ferric Reducing Antioxidant Power
**GA**	Gallic Acid
**HDL**	High-Density Lipoprotein
**IC_50_**	Inhibition Concentration of 50%
**iNOS**	Inducible Nitric Oxide Synthase
**IZD**	Inhibition Zone Diameter
**LD**	Lethal Dose
**LDL**	Low-Density Lipoprotein
**MBC**	Minimum Bactericidal Concentration
**MIC**	Minimum Inhibitory Concentration
**RNS**	Reactive Nitrogen Species
**ROS**	Reactive Oxygen Species
**SGOT**	Glutamooxaloacetate Transferase Serum
**SGPT**	Glutamate Pyruvate Transaminase Serum
**STZ**	Streptozotocin-Induced Diabetic
**TG**	Triglyceride
**TNF-α**	Tumor Necrosis Factor
**VLDL**	Very Low-Density Lipoprotein
